# White Coat Effect: Is It Because of the Hospital Setting, or Is It Physician-Induced?

**DOI:** 10.7759/cureus.38144

**Published:** 2023-04-26

**Authors:** Manvir Singh, Navpreet Singh, Hardik Pahuja, Rashmi Arora, Anshul ., Hardik Patel

**Affiliations:** 1 Internal Medicine, Dayanand Medical College and Hospital, Ludhiana, IND; 2 Internal Medicine, Gian Sagar Hospital & Medical College, Rajpura, IND; 3 Psychiatry, Gian Sagar Hospital & Medical College, Rajpura, IND; 4 Anesthesiology and Critical Care, Pandit Bhagwat Dayal Sharma Post Graduate Institute of Medical Sciences, Rohtak, IND; 5 Anesthesiology, Pandit Bhagwat Dayal Sharma Post Graduate Institute of Medical Sciences, Rohtak, IND; 6 Urology, Medanta, Gurugram, IND

**Keywords:** physician effect, hospital effect, masked effect, white coat effect, ambulatory blood pressure monitoring, white-coat hypertension

## Abstract

Introduction: White coat hypertension (WCH) patients are those individuals who have high blood pressure (BP) in the medical environment but are normal during their daily activities. White coat hypertensive patients with normal daytime ambulatory blood pressure monitoring (ABPM) rapidly progress to sustained hypertension. WCH is mainly treated with non-pharmacological methods. Alpha-1 agonists and beta blockers are logical treatment choices for patients with fixed hypertension with the White Coat Effect (WCE). Masked hypertension patients are those individuals who have normal values at the doctor’s office but elevated BP at home or during 24-hour ABPM (24-hour or daytime). ABPM is a more practical and reliable method for detecting patients with WCH.

Material and methods: This observational study was conducted at Dayanand Medical College & Hospital, Ludhiana, over the course of one year (December 2015 to November 2016). The primary objective of the study was to determine whether there was a difference in blood pressure readings between the home setting and the hospital setting. The secondary objective was to determine whether the difference, if present, between the hospital and home readings was due to the hospital setting, physician presence, or a combination of both. Patients with stage 1 hypertension were included in the study, irrespective of antihypertensive treatment. Patients with ischemic heart disease, chronic liver failure, and chronic kidney disease who could not follow protocol instructions were excluded.

Results: In our study, the mean age of patients was 53.91±12.86 years. The patient’s mean systolic blood pressure (SBP) and diastolic blood pressure (DBP) readings at the hospital were higher than their home readings (p-0.012; p-0.001, respectively). Mean hospital SBP and DBP readings recorded by the physician were higher than readings recorded by patients alone at home (p-0.002; p-0.014, respectively) and alone at the hospital (p-0.004; p-0.001, respectively). BP readings taken by the physician with a manual sphygmomanometer were significantly lower than those taken with a digital sphygmomanometer by patients and physicians in all settings (p<0.05). The mean rise in BP was significant in both the physician's presence and the hospital environment (p<0.05 for both), and this rise was more significantly associated with the hospital effect than the physician effect (p<0.05).

Conclusion: Misdiagnosis of hypertension results in inappropriate prescription and overuse of antihypertensive medications for individuals who are not persistently hypertensive. So it is very important to rule out WCH in both the hospital setting and the physician's presence, more precisely by ABPM. WCH can be diagnosed with regular BP monitoring by a digital sphygmomanometer at home.

## Introduction

Hypertension is defined as resting blood pressure (BP) at or above 130/80 mmHg in adults [1]. White coat hypertension (WCH), also referred to as "office hypertension" [2] or "isolated clinical hypertension" [3], is a term used to denote individuals who have high BP in the medical environment but normal BP during their daily activities. It is considered clinically significant when office BP exceeds daytime ambulatory blood pressure monitoring (ABPM) by 20/10 mmHg, irrespective of antihypertensive treatment [4,5].

The white coat effect (WCE) is a measure of blood pressure change from before to during a visit to the office or clinic when the blood pressure is recorded by a physician or nurse. It is associated with peripheral sympathetic hyperactivity due to anxiety in the clinical setting. It is a prehypertensive state with pronounced inter-subject variability [6]. WCE increases the incidence of myocardial ischemia, microalbuminuria, and retinopathy, suggesting that long-term cardiovascular outcomes in WCH are intermediate between normotensive and sustained hypertensive subjects [7,8].

Masked hypertension patients are those individuals who have normal values at the doctor’s office but elevated BP at home or during 24-hour ABPM (24-hour or daytime) [4,9].

A sphygmomanometer is the most common non-invasive instrument used to measure BP. ABPM is the more practical and reliable method for detecting patients with WCH and predicting target organ damage. Multiple readings are recorded in a single day, with the advantage of nocturnal readings for analysis [10,11]. Home BP measurements can be used effectively to guide anti-hypertensive treatment [12].

Errors in BP measurement are a major source of BP misclassification. The failure to adequately diagnose white coat hypertension with standardized measurements has led to the inappropriate prescription and overuse of antihypertensive medications for individuals who are not persistently hypertensive [3]. Hence, we proposed to conduct this observational study to explore whether there is a difference in blood pressure readings between the home setting and hospital setting; if there is, whether this difference is due to the hospital setting, the physician's presence, or a combination of both. We hypothesized that blood pressure would be elevated in hospital settings.

## Materials and methods

This observational study was conducted at Dayanand Medical College and Hospital, Ludhiana, India, from December 2015 to November 2016, after approval by the institutional ethical committee vide letter no. DMCH/4/15-2015 and informed written consent from patients. Seventy-five outpatient department (OPD) patients with stage 1 hypertension, i.e., BP=140-159/ 90-99 as per the Seventh Report of the Joint National Committee on Prevention, Detection, Evaluation, and Treatment of High Blood Pressure (JNC 7) Adult Classification of Hypertension [13], irrespective of antihypertensive treatment, were included in the study. BP was recorded by the physician during three different office visits with a digital sphygmomanometer. Patients with ischemic heart disease, chronic liver failure, and chronic kidney disease who could not follow protocol instructions were excluded.

A predefined proforma was used as a tool for data collection. The symptoms, signs, treatment history, and investigations related to hypertension, i.e., serum electrolytes, serum creatinine, and determination of estimated glomerular filtration rate (GFR), urine microalbumin-to-creatinine ratio, fasting blood glucose and lipogram, 12-lead electrocardiogram (ECG) [14], and other comorbidities, were recorded.

The procedure for BP measurement with a digital sphygmomanometer was demonstrated. All patients were instructed to note their BP readings on a performa sheet twice daily for six consecutive days. The patients were advised to take the medications after recording their BP readings. The readings were recorded at 7 am and 7 pm, each day. They were instructed to rest their right arm on a table at heart level and place an appropriate-sized cuff. The size of the cuff was decided on the basis of the patient's arm circumference. [13] Recruited patients were provided a digital sphygmomanometer with an appropriate-size cuff for six days. Tea, coffee, alcohol, and cigarettes were to be avoided 30 minutes before recording.

On the seventh day, patients were instructed to visit the hospital at one of the times they were taking their home blood pressure readings, and additional blood pressure readings were noted there. One reading was taken by the patient themselves using a digital machine after a rest of three minutes in an empty room. Another reading was taken by the physician using a digital machine after a rest of three minutes. After another three minutes, the clinician recorded the next reading using a manual sphygmomanometer.

We also assessed the magnitudes of the hospital and physician effects in determining the WCE and compared both effects. A higher BP recorded by the physician in the hospital than home BP was regarded as WCE, whereas a lower BP recorded by the physician in the hospital than home BP was regarded as masked effect. The hospital effect was a higher BP recorded by the patient in the hospital than at home. The WCE minus the hospital effect was considered the physician's contribution to the BP differences (the physician effect).

Statistical Analysis

The prevalence rate of hypertension (HTN) in Punjab is 17.5% based on previous data [15]. Assuming this as the reference value, the minimum required sample at a 95% confidence level and ±9% precision worked out to 68. This number had been increased to 75 to allow for a predicted dropout from the study. For the estimation of sample size, the following formula has been used:

n=(Z2ɑ ✕ P ✕ (1-P))/d2 where Zɑ = the value of standard normal variate corresponding to ɑ level of significance; P = the likely value of the parameter; Q = 1-P; D = margin of error, which is a measure of precision.

Data were described in terms of range, mean ±standard deviation (± SD), median, frequencies (number of cases), and relative frequencies (percentages) as appropriate. A comparison of quantitative variables was made using paired t-tests for parametric data. For comparing categorical data, the Chi-square (χ2) test was performed, and Fisher's exact test was used when the expected frequency was less than five. A probability value (p-value) less than 0.05 was considered statistically significant. All statistical calculations were done using IBM Statistical Package for Social Science (SPSS 21), version 21 for Microsoft Windows.

## Results

Out of 75 subjects, 49 were male and 26 were female. The mean age was 53.91±12.86 years, with the maximum number (28%) in the age group of 61-70 years. The mean height was 165.95±6.81 cm, the mean weight was 74.16±10.51 kg, and the mean body mass index (BMI) was 27.11±3.36 kg/m2 (Table 1).

**Table 1:** Patient characteristics cm: centimeter; kg: kilograms; Std. deviation: standard deviation

	No of patients	Minimum	Maximum	Mean	Std. deviation
Age (years)	75	30.00	80.00	53.91	12.86
Height (cm)	75	144.00	178.00	165.95	6.81
Weight (kg)	75	53.00	99.00	74.16	10.51
BMI (kg/m^2^)	75	21.00	34.90	27.11	3.36

**Figure 1:**
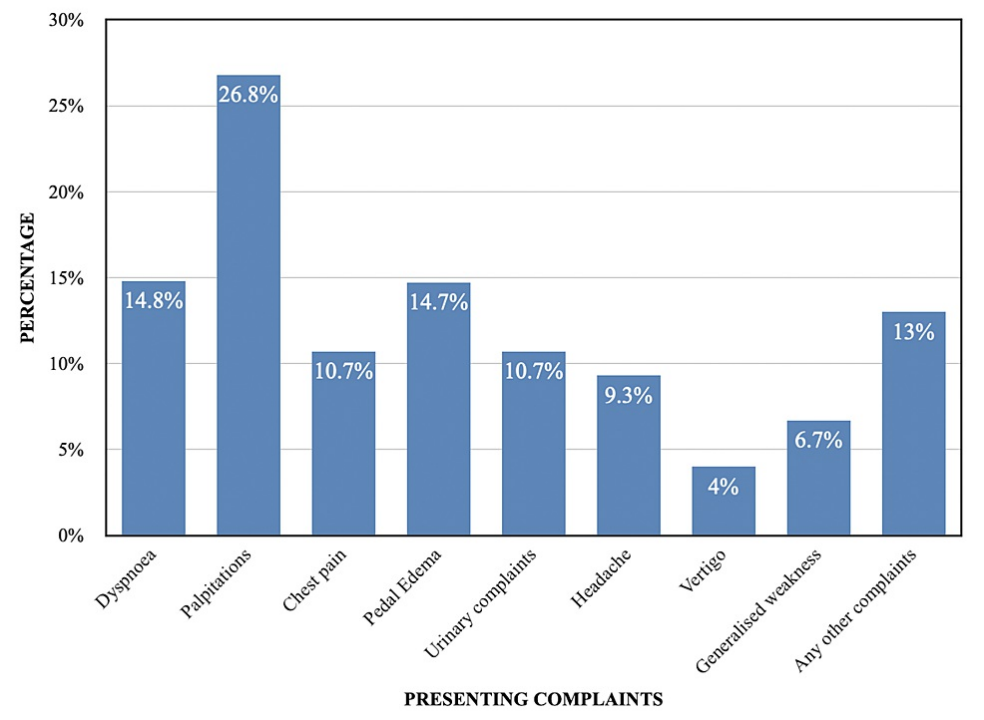
The distribution of patients according to complaints on presentation

Palpitation was the most common presenting complaint, followed by dyspnea and pedal edema (Figure 1).

Hypertension was prevalent in 28.1% of the patients who had it for one to five years (Table 2).

**Table 2:** The distribution of patients according to the duration of hypertension

Duration of hypertension	No. of patients	Percentage
0- 6 months	19	25.3%
6-12 months	10	13.3%
1-5 years	21	28.1%
5-10 years	16	21.3%
More than 10 years	9	12.0%
Total	75	100.0%

In this sample, 64% of patients were non-diabetics; 66.7% of patients had concentric left ventricular hypertension (LVH) on an electrocardiogram (ECG); and 53.3% of patients had cardiomegaly on a chest x-ray. GFR was calculated using the Chronic Kidney Disease Epidemiology Collaboration (CKD-EPI) equation [16]. Mildly decreased eGFR (60-89 ml/min/1.73 m2) was seen in 37.2% of patients, mildly to moderately reduced eGFR (45-59 ml/min/1.73 m2) in 23.5%, and 7.8% of patients had moderately to severely decreased eGFR (30-44 ml/min/1.73 m2).

Mean hospital systolic blood pressure (SBP) and diastolic blood pressure (DBP) (142.51±7.07/91.04±4.11mmHg) measured by the patients alone were significantly higher than mean home SBP and DBP (140.80±5.15/89.68±3.38 mmHg) measured by the patients using a digital sphygmomanometer (p-0.012; p-0.001, respectively) (Table 3).

**Table 3:** Mean BP readings BP: blood pressure; SBP: systolic blood pressure; DBP: diastolic blood pressure

BP readings	No. of patients	Minimum (mm Hg)	Maximum (mm Hg)	Mean (mm Hg)	Std. deviation (mm Hg)
SBP recordings by patients with a digital sphygmomanometer at home	75	126.17	151.08	140.80	5.15
DBP recordings by patients with a digital sphygmomanometer at home	75	81.58	98.00	89.68	3.38
SBP recordings by the patients with a digital sphygmomanometer in the hospital	75	128.00	170.00	142.51	7.07
DBP recordings by the patients with a digital sphygmomanometer in the hospital	75	80.00	102.00	91.04	4.11
SBP recordings by the physician with a digital sphygmomanometer in the hospital	75	128.00	177.00	146.76	8.19
DBP recordings by the physician with a digital sphygmomanometer in the hospital	75	84.00	110.00	94.44	5.27
SBP recordings by the physician with a manual sphygmomanometer in the hospital	75	120.00	160.00	136.53	6.61
DBP recordings by a physician with a manual sphygmomanometer in the hospital	75	74.00	98.00	85.73	4.23

The mean difference in this group was 1.71 ± 5.76/ 1.36 ± 3.52 mmHg (Table 4).

**Table 4:** Mean differences in BP in various settings with a digital sphygmomanometer SBP: systolic blood pressure, DBP: diastolic blood pressure

	No of patients	Minimum difference (mm Hg)	Maximum difference (mm Hg)	Mean difference (mm Hg)	Std. deviation (mm Hg)
SBP: Patient at home vs. Patient in the hospital	75	-8.00	33.67	1.71	5.70
DBP: Patient at home vs. Patient in the hospital	75	-6.33	10.67	1.36	3.52
SBP: Patient at home vs. Physician in the hospital	75	-12.00	40.67	5.96	6.92
DBP: Patient at home vs. Physician in the hospital	75	-3.42	18.33	4.76	4.57
SBP: Patient at home vs. Physician in the hospital	75	-4.00	14.00	4.25	3.80
DBP: Patient at home vs. Physician in the hospital	75	-4.00	12.00	3.40	3.28

The mean hospital SBP and DBP (146.76±8.19/94.44 ±5.27 mmHg) measured by the physician were significantly higher than the mean home SBP and DBP (140.80±5.15/89.68±3.38 mmHg) measured by the patients using a digital sphygmomanometer (p-0.002 and p-0.014, respectively). The mean difference in this group was 5.96 ± 6.92/ 4.76 ± 4.57 mmHg.

The mean hospital SBP and DBP (146.76±8.19 /94.44 ±5.27 mmHg) measured by the physician were significantly higher than the mean hospital SBP and DBP (142.51±7.07/91.04±4.11 mmHg) measured by the patients alone using a digital sphygmomanometer (p-0.004 and p-0.001, respectively). The mean difference in this group was 4.25 ± 3.80/ 3.40 ±3.28 mmHg.

The mean rise in BP due to the physician's presence in the hospital vs. the home environment was 7.6±6.4/5.6±4.2 mmHg; the mean increase in BP in the hospital vs. the home environment was 5.1±3.1/4.5±2.5 mmHg (p<0.05 for both).

The mean decrease in BP in the home environment vs. the physician's presence in the hospital was 3.0 ± 2.5/2.1 ± 0.9 mmHg, and in the hospital vs. the home environment, it was 2.5 ± 2.0/2.4±1.7 mmHg (p<0.05 for both).

When measured with a digital sphygmomanometer, no significant blood pressure difference was present between mean morning and mean evening SBP and DBP readings taken at home (p-0.334, p-0.096, respectively).

Comparing the mean SBP and DBP readings taken by patients with a digital sphygmomanometer at home vs. the mean SBP and DBP readings taken by the physician at the hospital with a manual sphygmomanometer, the manual readings were significantly lower than the home readings (p-0.004 and p-0.002, respectively).

Comparing the mean SBP and DBP readings taken by patients with a digital sphygmomanometer at the hospital vs. the mean SBP and DBP readings taken by the physician with a manual sphygmomanometer at the hospital, the manual readings were significantly lower than the patient readings (p -0.002 and p-0.014, respectively).

Compared to hospital digital vs. manual mean SBP and DBP readings taken by the clinician, manual readings were significantly lower than digital readings (p-0.012 and p-0.001, respectively).

## Discussion

Traditional solitary office BP measurements (OBPM) have a limited role in adequately diagnosing hypertension. Out-of-office BP measurements like ABPM and self-BP measurements (SBPM) should be obtained to guide the diagnosis and management of hypertension [17].

Our demographic data is consistent with Ahmet Adiyaman et al.'s [18] study, in which the mean age was reported as 55±13 years and the mean BMI was 27.6±4.6 kg/m2.

In our study, 66.7% of patients had concentric LVH on ECG. In a study by Cesare Cuspidi et al., the average prevalence of LVH was 40% [19], while Isaac W. Hammond et al. found LVH in 32% of patients [20].

Our study demonstrates that mean BP readings at home were lower than hospital mean BP readings measured by patients (p < 0.05), confirming our hypothesis that the hospital environment significantly contributes to the increase in BP readings compared to the home BP readings. Similar observations were made by Gerin W et al. and Stergiou GS et al., who concluded that the hospital environment plays a significant role in the white coat effect [21,22]. A study done by Isam Al-Karkhi et al. also demonstrated that the mean self-measurement by the patient at the office (SMOBP) was higher than self-home measurements [23]. However, our results were incongruent with the results reported by MA Young et al., who observed that both SBP and DBP were consistently higher at home, and this effect was independent of the presence of an observer [24].

The mean home BP readings by patients were lower than the mean BP readings by the physician with the digital sphygmomanometer in the hospital (p<0.05). This shows that the physician’s presence significantly contributes to the increase in BP readings. Similar observations were made by Martin G Myers et al., who observed that BP recorded in the examining room using an automated device by patients was similar to the mean awake ambulatory blood pressure (ABP), with both values being lower (p < 0.001) than the BP recorded on a routine visit to the patient‟s own family physician [25]. A study done by Stergiou GS et al. and Isam Al-Karkhi et al. also demonstrated similar results, concluding that a physician's presence plays a significant role in the white coat effect [22,23].

The mean hospital BP readings by patients alone were lower than the mean hospital BP readings by the physician with the digital sphygmomanometer (p<0.05), implying that the physician's presence in the hospital environment further accentuates the increase in BP readings contributed by the hospital environment. Similar observations were made by Martin G Myers and Miguel A Valdivieso [26].

In our study, the mean BP (WCE-BP) rise in the home environment vs. physician presence in the hospital was 7.6±6.4/5.6±4.2 mmHg. The contribution of WCE due to the hospital environment was 5.1±3.1/4.5±2.5 mmHg, and due to a physician's presence it was 2.5±1.6/3.3 ± 1.5 mmHg, so it was concluded in the study that the white coat effect was more due to the hospital environment as compared to physician presence (p< 0.05). Similar observations were made by Xi-xing Wang et al., who concluded that the white coat effect was more due to the hospital environment as compared to a physician's presence [27].

The masked effect of BP in our study in the home environment vs. the physician's presence in the hospital was 3.0 ± 2.5/2.1 ± 0.9 mmHg. The contribution of the masked effect due to the hospital environment was 2.5 ± 2.0/2.4±1.7 mmHg, and the contribution due to the physician's presence was 0.5 ±0.1/0.1 ± 0.02mmHg. Thus, it was concluded that the masked effect was more due to the hospital environment as compared to the physician's presence (p< 0.05). A study done by Ahmet Adiyaman et al. also demonstrated similar results, concluding that the masked effect consisted of a substantially larger hospital effect than the physician effect [18].

BP readings recorded with a manual sphygmomanometer by a physician were significantly lower than those recorded with a digital sphygmomanometer. Our results were inconsistent with those of Muniyandi M et al [28]. The difference between manual readings and the readings recorded by a digital sphygmomanometer was possibly due to the use of different principles. Hence, the findings observed with the manual sphygmomanometer cannot be given significant importance for understanding the effect of hospital and physician presence on blood pressure in our study.

It was concluded that the blood pressure differences between home and hospital settings were due to the hospital environment and physician presence. ABPM readings are close to the patient’s actual BP. Therefore, ABPM is an effective measure to reduce the white coat effect.

There are certain limitations to our study. Our study is a single-center study with a relatively smaller sample size. Also, the effect of a physician's empathy, which is significantly associated with a decrease in WCE, is not considered in this study. Future multicentric studies involving various patient cohorts to study the white coat effect and the masked effect are warranted.

## Conclusions

WCE can lead to the misclassification of normotensive subjects as having stage 1 or stage 2 hypertension. This misdiagnosis can result in unnecessary lifelong treatment with antihypertensive medication, which have potential side effects that may be seriously debilitating, especially in the elderly. Moreover, failure to identify the condition results in a large expenditure on unnecessary drugs. Therefore, WCH should be ruled out first in both the hospital setting and physician presence, more precisely by ABPM.
